# Food poisoning from raw horse meat contaminated with Shiga toxin-producing *Escherichia coli* O157 linked to nationwide spread of closely related strains, Japan, 2023

**DOI:** 10.1128/spectrum.04115-25

**Published:** 2026-03-31

**Authors:** Junji Seto, Kenichi Lee, Yohei Matoba, Hidemasa Izumiya, Mayu Suzuki, Tatsuya Ikeda, Katsumi Mizuta, Yo Sugawara, Motoyuki Sugai, Yukihiro Akeda, Sunao Iyoda

**Affiliations:** 1 Yamagata Prefectural Institute of Public Health 74178, Yamagata, Japan; 2 Department of Bacteriology I, National Institute of Infectious Diseases, Japan Institute for Health Security 13511 , Tokyo, Japan; 3 Antimicrobial Resistance Research Center, National Institute of Infectious Diseases, Japan Institute for Health Security 13511 , Tokyo, Japan; National University of Singapore, Singapore, Singapore

**Keywords:** Shiga toxin-producing *Escherichia coli*, STEC, O157, raw horse meat, multilocus variable-number tandem-repeat analysis, whole-genome sequencing, field epidemiology

## Abstract

In August 2023, 74 symptomatic patients developed Shiga toxin-producing *Escherichia coli* (STEC) O157 infection after consuming raw horse meat processed at a meat shop in Yamagata, Japan. To investigate the outbreak, we conducted multilocus variable-number tandem-repeat analysis (MLVA), whole-genome sequencing (WGS), and a nationwide retrospective epidemiological survey. MLVA and WGS identified 165 genetically similar strains collected across Japan during 2022–2023. Epidemiological investigations revealed that 6/89 (6.9%) cases in 2022 and 49/76 (64.5%) cases in 2023 were linked to raw horse meat consumption from the shop. WGS of 147 strains revealed a central genomic cluster of 76 strains, while others differed by 1–8 single-nucleotide variants from this cluster. The central genomic cluster contained strains from 2022 and 2023 obtained from patients who consumed raw horse meat. Epidemiological links and clonality of O157 strains suggested continuous contamination at the shop since 2022. Indeed, the shop lacked proper hygiene management based on hazard analysis and critical control point principles and exhibited poor sanitary conditions; however, direct evidence of the causative facility was not obtained because the suspected contaminated raw horse meat could not be sampled. This preliminary investigation highlights the importance of strict hygiene management in facilities producing meat for raw consumption and demonstrates the value of combining nationwide molecular epidemiology with field investigations to identify STEC outbreak sources.

**IMPORTANCE:**

Investigations of Shiga toxin-producing *Escherichia coli* (STEC) foodborne outbreaks incorporating whole-genome sequencing (WGS) have not been widely conducted to date. This study demonstrated that the combination of multilocus variable-number tandem-repeat analysis, WGS, and nationwide surveillance allowed for the detection of a dispersed STEC O157 outbreak linked to raw horse meat. Simultaneously, the investigation suggested persistent and low-diversity contamination at a single meat shop over a 1-year period. This study highlights (i) how combining genomic analysis with epidemiological investigation can identify geographically widespread STEC cases, (ii) the risk posed by inadequate hygiene management in facilities producing meat for raw consumption, and (iii) the potential for STEC strains to persist with minimal genomic mutations in food or processing environments. These insights support targeted control measures such as improved sanitation, the implementation of hazard analysis and critical control point principles, and ongoing genomic surveillance to prevent similar outbreaks.

## INTRODUCTION

Shiga toxin-producing *Escherichia coli* (STEC) is a foodborne or waterborne pathogen that can cause diarrhea and life-threatening hemolytic uremic syndrome (HUS) (1, 2). It can also spread through secondary human-to-human transmissions. Of the 185 STEC serogroups identified at the Statens Serum Institut (3), O157 is the most frequently detected in humans (1). Shiga toxin (Stx) is the principal virulence factor of STEC, and strains carry Stx1, Stx2, or both (1). STEC isolates producing Stx2 are more pathogenic than those producing Stx1 (1). The stx genes are transferred horizontally by bacteriophages but may be lost within the human body or under environmental conditions (4–6).

Ruminants are the primary STEC reservoir, and contaminated beef is a major source of STEC transmission (6–9). In contrast, its prevalence in non-ruminants is low (10, 11). For instance, STEC is rarely detected in horses unless co-housed with ruminants (12, 13). In Japan, raw meat consumption has long been practiced. Raw beef processed under strict controls is currently available in markets, following a large-scale outbreak linked to the raw beef dish yukhoe (6). However, raw horse meat production is subject only to general hygiene measures and not specific legislation. Consumers typically eat sliced raw horse meat, sold refrigerated with soy sauce and condiments. Therefore, contamination of raw horse meat with pathogens directly leads to foodborne illness. Two outbreaks associated with raw horse meat have been documented in Japan: an O157 outbreak from domestic products in 2014 and O26 infections from imported products in 2023 (14, 15). In these cases, STEC was isolated from raw horse meat, and the multilocus variable-number tandem-repeat analysis (MLVA) profiles were consistent with those of the patient isolates; however, the source of contamination remains unclear.

Molecular epidemiology can provide critical insights into the sources of outbreaks (2, 8). MLVA, which examines microsatellite regions of STEC, is a rapid screening tool for identifying genetically related strains (2, 8, 16). In Japan, municipal public health institutes generate MLVA profiles for major serotypes such as O157, which are centralized at the National Institute of Infectious Diseases (NIID) for nationwide comparison. Although whole-genome sequencing (WGS) is not routinely employed in ongoing outbreak investigations, NIID is developing genomic archives of preserved STEC isolates. A threshold of ≤10 single-nucleotide variants (SNVs) has been proposed to define closely related *E. coli* strains (17), yet data on SNV distributions in STEC outbreaks remain limited (18–20).

This study presents a nationwide retrospective investigation of a food poisoning case caused by raw horse meat in Japan in 2023. It integrates nationwide comparisons of MLVA, WGS, and epidemiological survey results collected between 2022 and 2023.

## MATERIALS AND METHODS

### Investigation of a food poisoning case

In August 2023, an STEC O157 food poisoning case was investigated by public health centers in Japan under the Food Sanitation Act and the Infectious Diseases Control Law. In Yamagata Prefecture, staff of four public health centers investigated symptomatic patients after consuming raw horse meat from a meat shop (Shop A) in Yamagata. While the investigation methods in municipalities outside Yamagata were unclear, information on patients who became ill after consuming raw horse meat at Shop A was shared with the Yamagata Prefectural Government. The case definition included individuals who developed symptoms in August 2023 after consuming raw horse meat processed at Shop A.

### Culture of O157

Stool specimens from patients and their family members, as well as Shop A staff and their family members, were directly inoculated onto MacConkey Agar (Becton, Dickinson and Co., Franklin Lakes, NJ, USA) with lactose, MacConkey Agar with sorbitol and Cefixime Tellurite Selective Supplement (Oxoid, Ltd., Hampshire, UK), and CHROMagar STEC (Kanto Chemical Co., Inc., Tokyo, Japan), and incubated at 36°C for 22 h. Simultaneously, specimens suspended in modified EC broth with novobiocin (Kyokuto Pharmaceutical Industrial Co., Ltd., Tokyo, Japan) and incubated at 42°C for 22 h were also inoculated onto these agar media and incubated. For swab and raw horse meat samples from Shop A, an emulsion was prepared by adding ninefold volume of modified EC broth (Shimadzu Diagnostics Corp., Tokyo, Japan) and then incubated at 42°C for 22 h. These incubated samples were inoculated onto these agar media (i) directly and (ii) after concentration using immuno-magnetic beads for O157 (Denka Co., Ltd., Tokyo, Japan).

### MLVA

MLVA was performed for two purposes: (i) to compare profiles among food poisoning-related isolates identified in Yamagata in 2023 and (ii) to identify genetically similar strains among approximately 33,000 STEC isolates collected across Japan during 2013–2023. MLVA was conducted as described by Izumiya et al. (2). The 17 loci excluding O157-10 were applied in this study. At the Yamagata Prefectural Institute of Public Health, PCR was performed using the Platinum Multiplex PCR Master Mix (ThermoFisher Scientific) according to the manufacturer’s instructions. The PCR conditions were as follows: 95°C for 2 min; 35 cycles of 95°C for 30 s, 60°C for 90 s, and 72°C for 60 s; with final extension at 72°C for 10 min. PCR products were separated using a SeqStudio Genetic Analyzer (ThermoFisher Scientific). At the NIID, the Qiagen Multiplex PCR Plus Kit (Qiagen) was also used for PCR amplification, and an Applied Biosystems 3500xl Genetic Analyzer (ThermoFisher Scientific) was used for the separation of PCR products. The band sizes were determined using GeneMapper software and converted to repeat copy numbers. The null allele (no amplification product detected) was designated as −2. An MLVA complex was defined as isolates that were single-locus variants of the most common 17-locus profile, as well as isolates that differed at a single locus from those variants. A minimum spanning tree of MLVA complex strains was generated using BioNumerics v.7.6.3 (Applied Maths, Sint-Martens-Latem, Belgium).

### WGS and comparative genomics

WGS was conducted to detect strains closely related to the outbreak isolates, as previously described (21, 22). Specifically, genomic DNA was extracted using the MagMAX DNA Multi-Sample Ultra 2.0 Kit (ThermoFisher) with the KingFisher Duo Prime instrument. Genomic DNA libraries were prepared using the QIAseq FX DNA Library Kit (Qiagen) and sequenced as multiplexed paired-end reads (150-mer × 2) on NovaSeq (Illumina). FASTQ sequences and assembled contigs were deposited in the DNA Data Bank of Japan (http://www.ddbj.nig.ac.jp) under the BioProject number PRJDB23685. Short reads were assembled using SPAdes v.3.14.0 with the “--careful” option (23). Core genome SNV-based phylogenetic relationships were inferred using the SNPcaster pipeline (https://github.com/leech-rr/SNPcaster) (21, 22), with STEC O157 strain Sakai (GenBank accession no. BA000007.3) as the reference genome. Repetitive regions >50 bp were identified by MUMmer v.4.0.0 (nucmer, repeat-match, and exact-tandems functions) (24) and excluded from further analyses, as were prophage regions. Recombination-prone regions were identified using Gubbins v.3.4 (24) and removed. The remaining concatenated SNV sequences were used for subsequent analyses. Median joining networks were constructed and visualized using PopART (25). Following previous reports (8, 26), strains with ≤10 pairwise SNV differences were considered to be closely related. Comparative analyses were performed using the in-house NIID WGS database, which contained approximately 10,000 STEC isolates collected nationwide between 2005 and 2023.

### Definition of genetically similar strains

Genetically similar strains were defined as O157 isolates that either formed an MLVA complex or were classified as closely related to the Yamagata outbreak isolates.

### Nationwide epidemiological survey

We investigated STEC O157 cases nationwide whose isolates were assigned as genetically similar strains. An anonymized case list was obtained from the National Epidemiological Surveillance of Foodborne Disease (NESFD), operated by the Ministry of Health, Labour and Welfare. The NESFD list includes information on patients reported by physicians nationwide for whom MLVA results were available (e.g., onset date, STEC isolation date, serotype, Stx type, MLVA type, and prefecture). Based on the list, Yamagata Prefectural Government requested municipalities to classify each patient into one of six groups. The response rate was 100% (Table S1). We defined patients assigned to “consumed raw horse meat from Shop A” or “secondary infection from a patient who consumed raw horse meat (later confirmed to be Shop A-related)” as the Raw-horse-meat group. The remaining four groups: “consumed raw horse meat from sources other than Shop A,” “consumed raw horse meat but the source of purchase was unknown,” “did not consume raw horse meat,” and “consumption of raw horse meat was not investigated” were defined as the non-Raw-horse-meat group.

### Statistical analysis

Fisher’s exact test was used to assess whether the distribution of Stx types and the proportion of Raw-horse-meat group differed between 2022 and 2023. The Wilcoxon rank sum test was applied to compare the SNV distributions between groups, under the hypothesis of differing genomic diversity. It should be noted that we counted the number of SNVs for each strain starting from the genome sequence that the outgroup strain initially reached in the median-joining network (central genomic cluster). Analyses were conducted with R v.4.4.3 (27), and statistical significance was set at *P* < 0.05.

## RESULTS

### Food poisoning case in 2023

In August 2023, 74 symptomatic patients with STEC O157 infection were identified after consuming raw horse meat from Shop A. Initially, two medical institutions reported to public health centers that patients developed symptoms such as bloody diarrhea after eating raw horse meat from Shop A. Subsequently, investigations by public health centers in Yamagata and information provided by municipalities outside Yamagata revealed new patients. Overall, patients who had consumed raw horse meat from Shop A were found in 42 family and other groups. Sixty patients resided in Yamagata, while 14 lived in four other prefectures. Thirty-one patients (41.9%) presented with bloody diarrhea. No patients developed HUS, and no deaths were reported. O157 was isolated from stool specimens of 26 of the 60 patients in Yamagata, whereas culture results from other prefectures are unavailable.

Concurrently, 15 O157 isolates were cultured from the family members in Yamagata.

The implicated batch of raw horse meat had already been sold when a public health center conducted an inspection at Shop A, 7 days after detecting the first patient. Four samples of raw horse meat from other batches and 20 swab samples of the shop collected during the inspection tested negative for STEC. Conversely, upon stool examination, O157 was isolated from one asymptomatic employee who was in charge of slicing raw horse meat. The shop, which sold only horse meat, also offered online sales outside Yamagata. Although connected to a public water supply, sanitary conditions were poor, with inadequate cleaning (e.g., dirty chilled-room walls and door handle, as well as use of heavily scratched and discolored cutting boards). Because self-initiated hygiene management based on hazard analysis and critical control point (HACCP) was not implemented in Shop A, employee health records were absent. Horses were raised in nearby stables and processed at a slaughterhouse certified for HACCP. At the slaughterhouse, cattle and horses were processed on the same line, but on different days. STEC was not detected from swab samples of the processing line examined after the outbreak (data not shown).

### MLVA in Yamagata

MLVA of the 26 isolates from patients, 15 from family members, and 1 from an employee revealed a single complex (41 indistinguishable and 1 single-locus variant profile).

### Comparison with molecular epidemiological data in Japan

**Fig 1:**
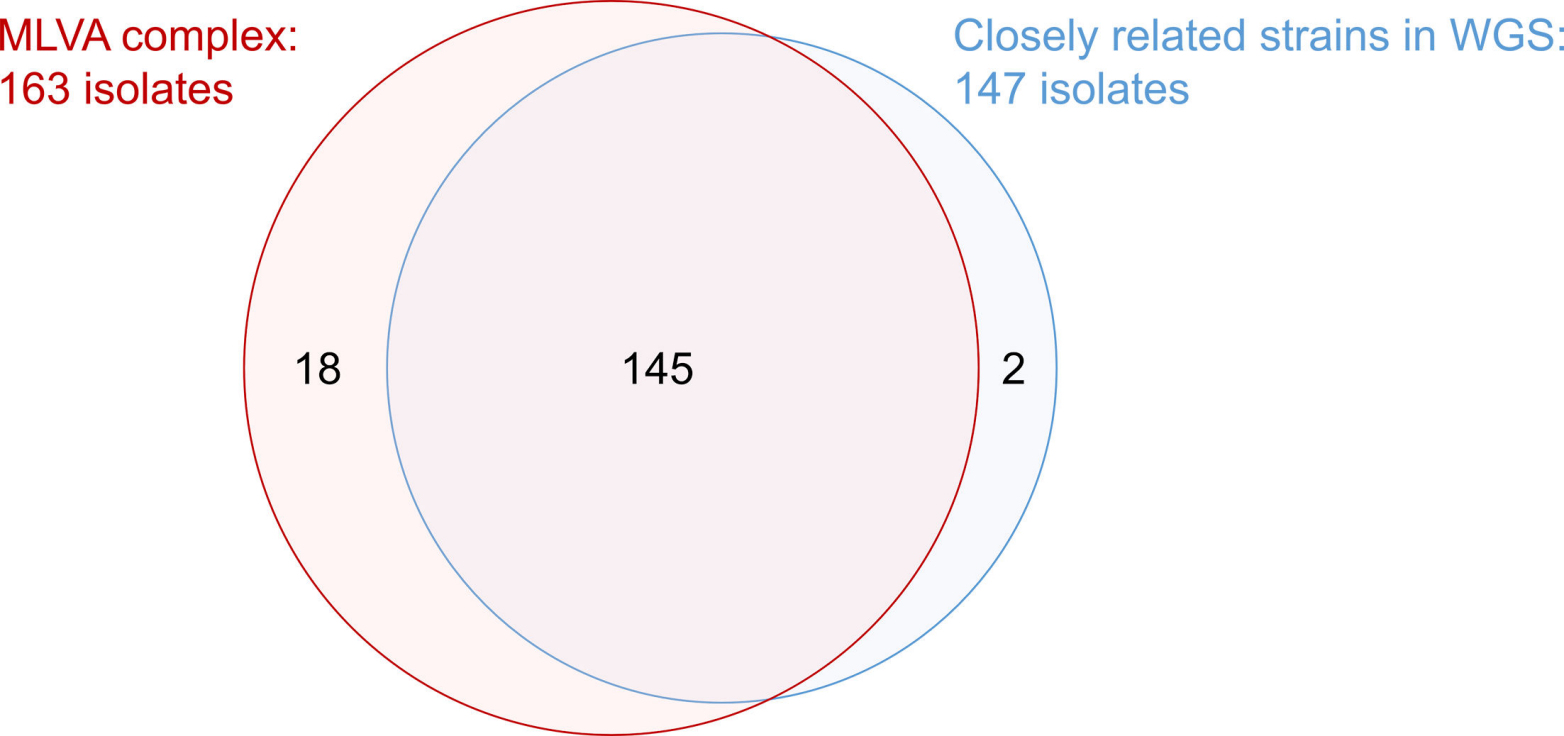
Venn diagram illustrating the overlap between 165 genetically similar O157 strains. Eighteen strains failed WGS. Two strains, which showed two-loci or four-loci variants from the most common profile, were confirmed as closely related strains through nationwide comparison of STEC isolates in Japan.

Figure 1 depicts the relationship between the 165 genetically similar strains. Comparison of MLVA profiles between Yamagata isolates and STEC isolates collected nationwide showed that 163 isolates during 2022–2023 formed an MLVA complex (Fig. S1). Of these, 145 strains underwent WGS and were classified as closely related strains. Two additional related strains were identified in the NIID in-house WGS database.

**Fig 2:**
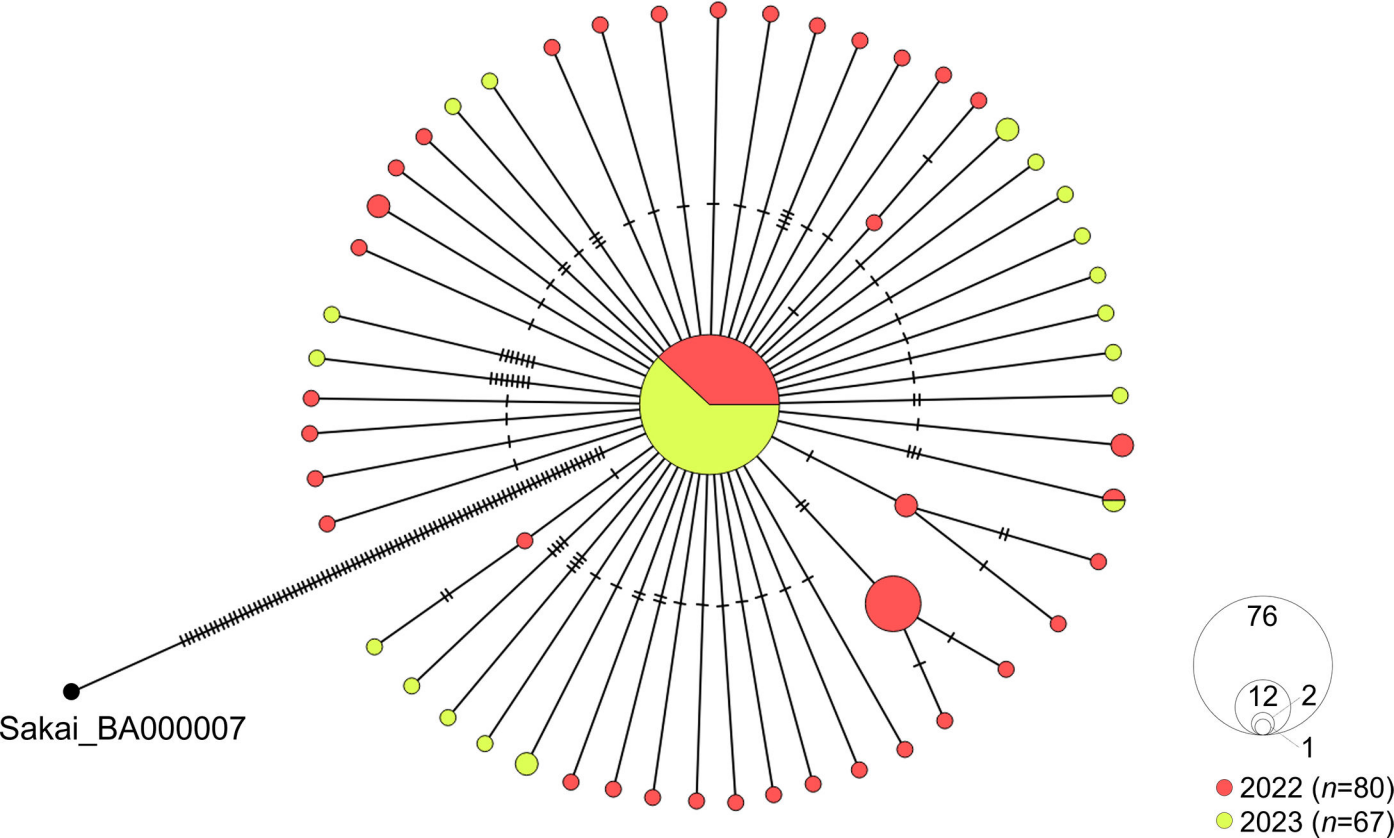
Median-joining network of 147 closely related strains of STEC, Japan, 2022–2023. Circles denote STEC strains, and hatch marks indicate the number of single-nucleotide variants.

A median-joining network of 147 strains revealed that 76 formed the largest cluster (Fig. 2). Others accumulated 1–8 genomic mutations, indicating clonal origin.

Stx typing of the 165 strains identified 109 (66.1%) as Stx1/2, 53 (32.1%) as Stx2, and 3 (1.8%) as Stx1. A statistically significant difference (*P* < 0.0001) in Stx distribution was observed between 2022 and 2023 (Table 1).

**TABLE 1:** Shiga toxin type in 165 STEC O157 strains, 2022–2023, Japan

	2022, *n* (%)	2023, *n* (%)
Stx1	3 (3.4)	0
Stx1/2	86 (96.6)	23 (30.3)
Stx2	0	53 (69.7)

### Nationwide epidemiological survey

**Fig 3:**
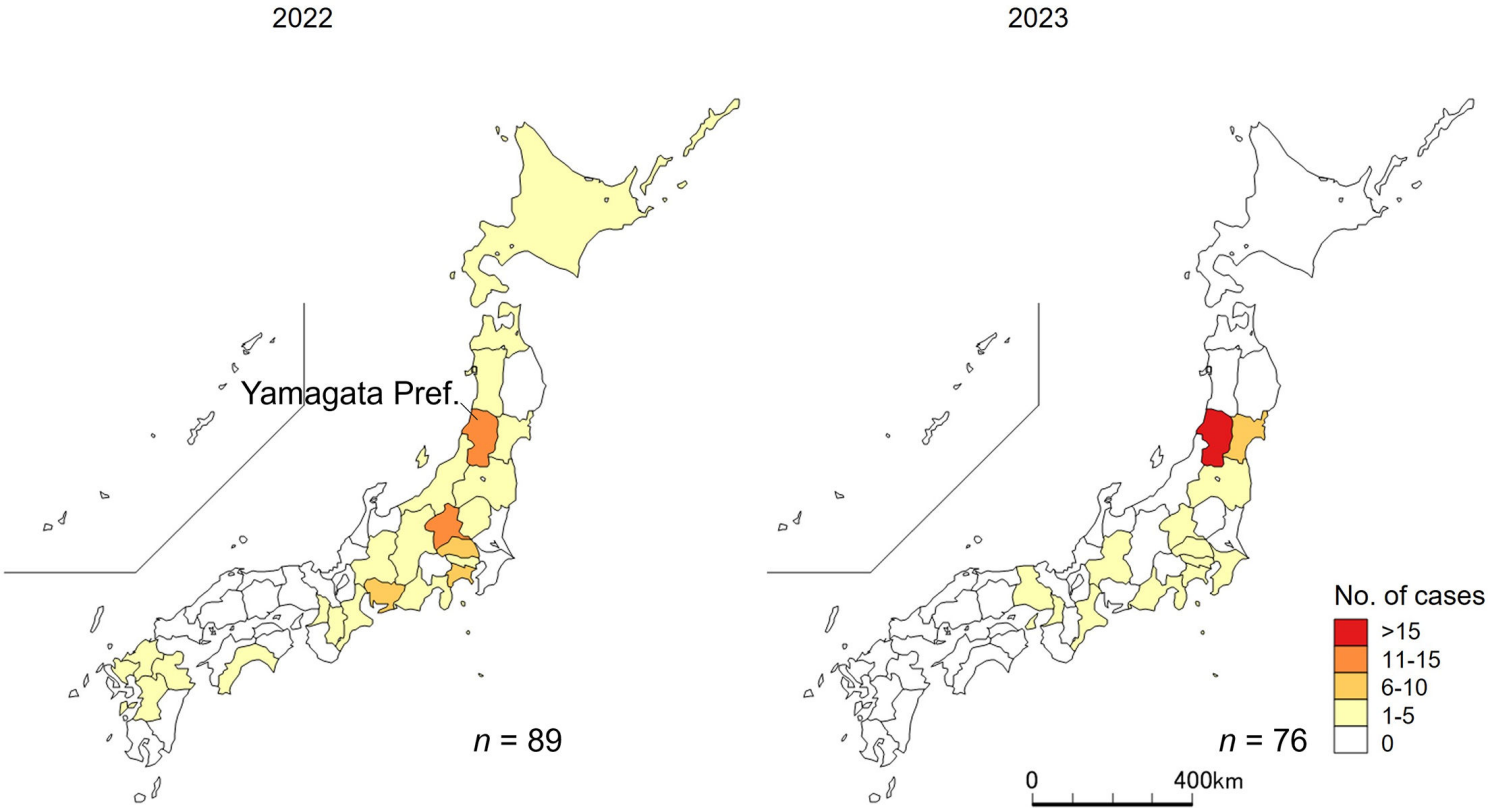
Geographical distribution of genetically similar Shiga toxin-producing *Escherichia coli* cases, Japan.

**Fig 4:**
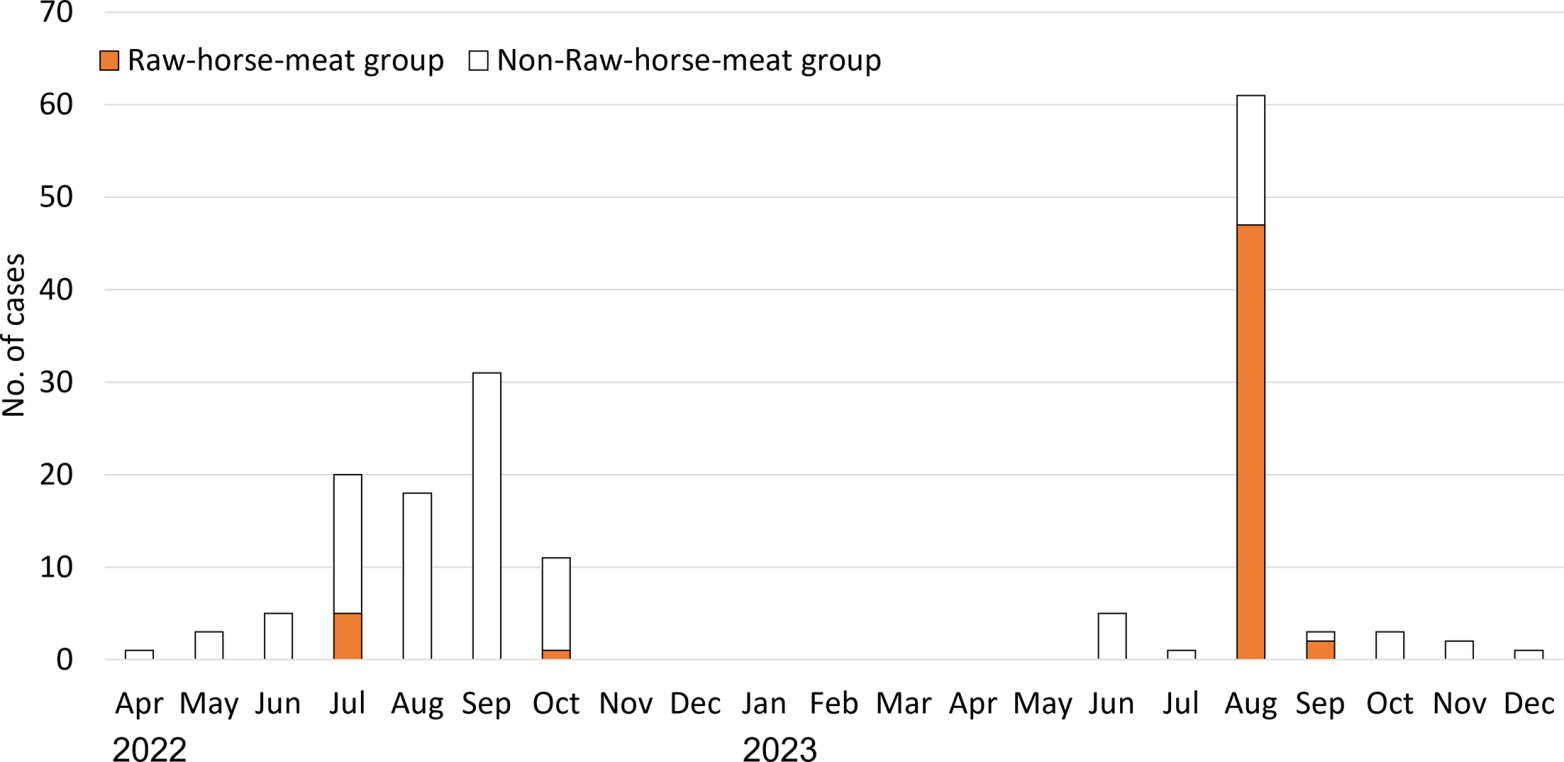
Temporal distribution of isolation month in 165 genetically similar Shiga toxin-producing *Escherichia coli* cases, Japan.

The 165 cases were distributed across 26 prefectures (Fig. 3). In 2022, 89 cases were reported from 24 prefectures. The distance from Yamagata, where Shop A is located, to the farthest prefecture was more than 1,000 km. In 2023, 76 cases were identified in 13 prefectures on the island of Honshu, with Yamagata reporting the highest number of 47 cases. Among the 165 cases, 6 in 2022 and 49 in 2023 were classified as belonging to the Raw-horse-meat group (Fig. 4). The proportion of the Raw-horse-meat group in 2022 was significantly lower than that in 2023 (6/89 [6.7%] vs 49/76 [64.5%], *P* < 0.0001). These findings suggest that raw horse meat from Shop A was not the principal driver of clonal spread in 2022.

### Characteristics of Raw-horse-meat group

**Fig 5:**
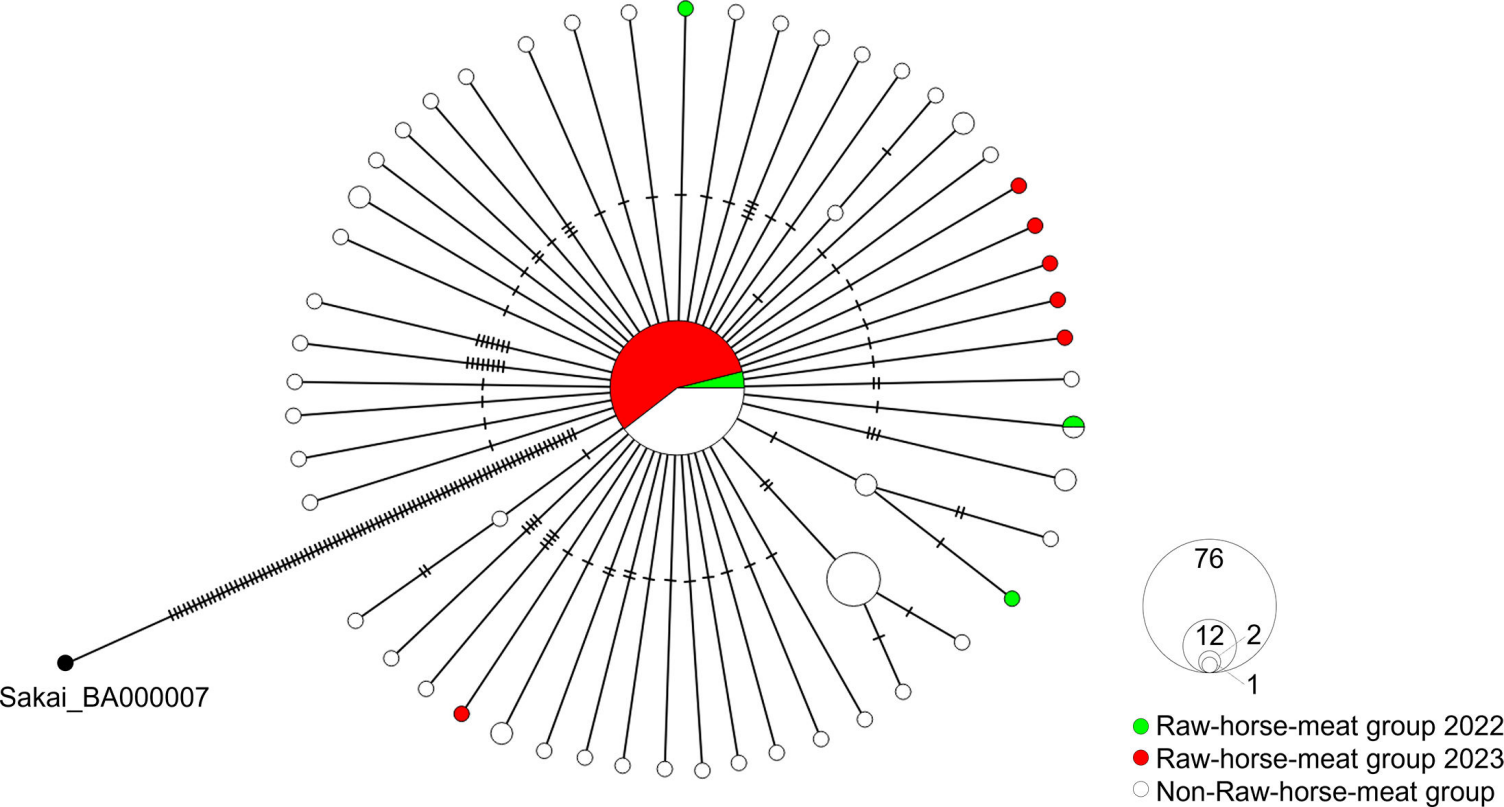
Median-joining network of 147 closely related strains of STEC, Japan, 2022–2023, incorporating results of nationwide epidemiological survey. Circles denote STEC strains, and hatch marks indicate the number of single-nucleotide variants.

**Fig 6:**
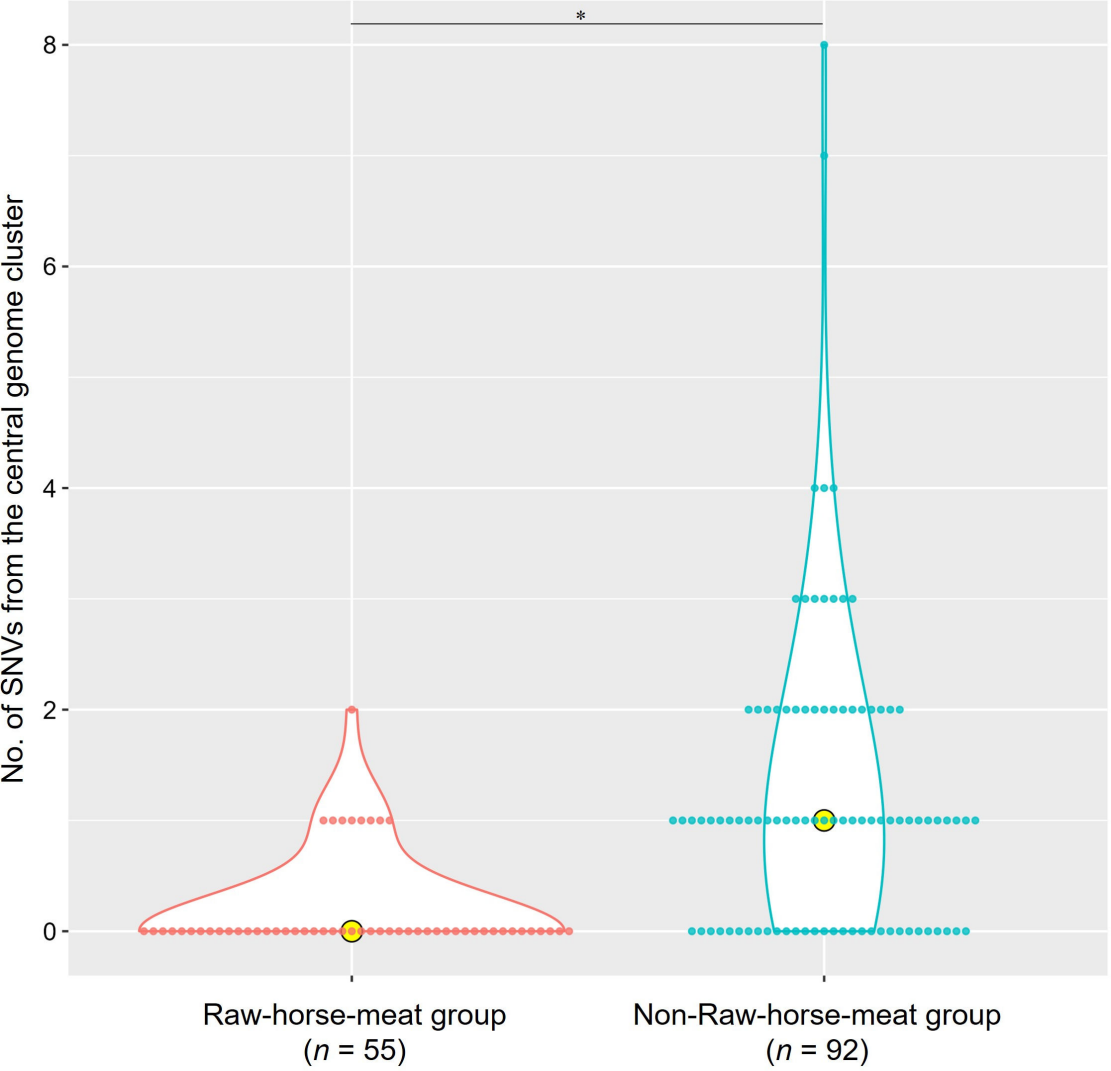
Comparison of Shiga toxin-producing *Escherichia coli* strains between the Raw-horse-meat and non-Raw-horse-meat groups. Dot and violin plots show SNVs from the central genomic cluster; yellow circles indicate medians. Asterisks denote *P* < 0.05.

In the median-joining network of the 147 strains, the largest genomic cluster included 46 Raw-horse-meat strains (Fig. 5). Notably, 3 strains in 2022 and 43 in 2023 were indistinguishable, indicating minimal genomic divergence over 1 year. The number of SNVs in this group ranged from 0 to 2, significantly fewer than in the comparison group (*P* < 0.0001) (Fig. 6).

Among 55 Raw-horse-meat strains, Stx2 predominated in 2023, coinciding with the large-scale outbreak (Table 2). The Stx distribution shifted significantly between 2022 and 2023 (*P* < 0.0001), with Stx2 replacing Stx1/2 as the dominant type.

**TABLE 2:** Shiga toxin type in 55 STEC O157 strains belonging to the Raw-horse-meat group

	2022, *n* (%)	2023, *n* (%)
Stx1	1 (16.7)	0
Stx1/2	5 (83.3)	1 (2.0)
Stx2	0	48 (98.0)

### Control measures

In October 2022, the local public health center advised Shop A to develop hygiene plans and records based on HACCP principles after detecting one O157 case linked to raw horse meat consumption. However, sanitary practices did not improve. Following the 2023 outbreak, hygiene management was strengthened substantially. As of September 2025, no additional cases have been linked to Shop A. Indeed, STEC has not been detected in raw horse meat after the shop reopened, and the maintenance of hygienic facility conditions has been confirmed through regular inspections by the local public health center. Furthermore, given that delays in sharing the information on sporadic cases among public health centers within Yamagata led to delays in sampling, a rapid information sharing system for STEC patients was established after the 2023 outbreak.

## DISCUSSION

To investigate the O157 food poisoning outbreak caused by raw horse meat in Japan in 2023, we conducted a nationwide survey integrating MLVA, WGS, and epidemiological investigations. The study revealed that (i) O157 isolates from patients who consumed raw horse meat from Shop A in 2023 were assigned to closely related strains by WGS, (ii) clonal O157 strains had been detected nationwide since 2022, and (iii) O157 strains associated with raw horse meat consumption in 2022 were also included in the closely related strains. These findings suggest that the underlying cause of the 2023 outbreak was the nationwide spread of the clonal O157 strains since the previous year. Furthermore, the clonality of raw horse meat-associated strains between 2022 and 2023 was consistent with continuous O157 contamination at Shop A since 2022, presumed from the inadequate hygiene management at the facility.

We could not identify the route of O157 contamination in Shop A. Because the prevalence of STEC in horses is low and Shop A raised only horses, it is unlikely that the horses carried O157 before slaughter (12). Previous STEC outbreaks linked to raw horse meat also had unclear contamination routes, suggesting secondary contamination (14, 15). Cross-contamination at the slaughterhouse was unlikely in this outbreak, as horses did not contact cattle during slaughter and STEC was not isolated from the HACCP-certified facility. A possible route was the introduction of O157 into Shop A through human carriers; however, further investigation was limited by the absence of health records for employees. This case demonstrates that horse meat can be contaminated with STEC, although the route is difficult to establish. Given that horse meat can also harbor *Salmonella* and *Yersinia enterocolitica*, strengthening hygiene management for raw meat remains essential (28, 29).

This preliminary investigation raises the possibility of paused genomic mutations in STEC. In this study, most strains in the Raw-horse-meat group did not accumulate mutations even after 1 year from initial detection. Accordingly, STEC may persist in food products or the environment without genomic change for extended periods. Supporting this hypothesis, the SNV count in the Raw-horse-meat group was significantly lower than that in the non-Raw-horse-meat group. Because bacterial genomes mutate during proliferation (30), refrigeration or freezing may contribute to slowed mutations, warranting further studies on STEC genomic stability under low temperatures.

The nationwide dissemination of closely related strains after 2022 remains unclear because the source of infection for the non-Raw-horse-meat group was not investigated. Given the smaller number of patients associated with raw horse meat and the detection of related strains in 2022, raw horse meat consumption is likely not the primary driver of nationwide dissemination. As of September 2025, Japan is establishing a nationwide investigation system for STEC food poisoning using the NESFD. Integrated investigations combining molecular and field epidemiology are expected to clarify the mechanisms of the nationwide spread of STEC.

Our study also examined the Stx variation. The distribution of Stx types in the Raw-horse-meat group shifted significantly from Stx1/2 to Stx2 during 2022–2023. Considering that Stx can be lost under prolonged storage at stable temperatures (5), the predominant O157 strain linked to Shop A may have shifted from Stx1/2 to Stx2. While the Stx type is important in outbreak investigations, it is noteworthy that Stx variation can occur in long-term cases.

This study has several limitations. First, we could not isolate O157 from Shop A or its products. Similarly, STEC contamination in Shop A’s horse stable was not investigated. Although the isolation of O157 from an employee indirectly supports contamination at Shop A, direct evidence of the causative facility was not obtained. Second, because the contamination route was not identified, independent contamination events in 2022 and 2023 cannot be excluded. Third, investigations of raw horse meat consumption were not performed for 20 of the 165 cases (Table S1), potentially underestimating the Raw-horse-meat group. Finally, WGS was not performed for the 18 strains forming the MLVA complex, leaving uncertainty about whether they represent closely related strains. However, given MLVA’s high discriminatory power for STEC, we included these strains (8). Importantly, statistical analyses restricted to the 147 closely related strains remained significant (data not shown).

In conclusion, this nationwide investigation showed that combining MLVA, WGS, and epidemiological surveillance enabled the detection of closely related strains in patients associated with raw horse meat consumption during 2022–2023. The 2023 outbreak could have been attributable to prolonged STEC contamination in the causative facility, underscoring the importance of strict hygiene management in facilities producing meat for raw consumption. Future studies with sampling of suspected contaminated food materials, standardized exposure assessments, and WGS of all related isolates are warranted to validate these findings and to clarify contamination sources, transmission routes, and strain relationships over time.

## Data Availability

FASTQ sequences and assembled contigs were deposited in the DNA Data Bank of Japan (http://www.ddbj.nig.ac.jp) under the BioProject number PRJDB23685. Individual accession numbers are listed in Table S1.

## SUPPLEMENTAL MATERIAL

Fig. S1 spectrum.04115-25-s0001.pdf Minimum spanning tree constructed from 163 Shiga toxin-producing *Escherichia coli* isolates, which formed a complex by 17 loci multilocus variable-number tandem-repeat analysis.

Table S1 spectrum.04115-25-s0002.xlsx Case list of Shiga toxin-producing *Escherichia coli* O157 infections, Japan, 2022-–2023.

ASM does not own the copyrights to Supplemental Material that may be linked to, or accessed through, an article. The authors have granted ASM a non-exclusive, world-wide license to publish the Supplemental Material files. Please contact the corresponding author directly for reuse.
