# Supplementary material for: Food poisoning from raw horse meat contaminated with Shiga toxin-producing Escherichia coli O157 linked to nationwide spread of closely related strains, Japan, 2023
Source: Microbiol Spectr. 2026 Mar 31;14(5):e04115-25. doi: 10.1128/spectrum.04115-25 (PMC13141936; doi:10.1128/spectrum.04115-25)
Supplement: Fig. S1 — Minimum spanning tree constructed from 163 Shiga toxin-producing Escherichia coli isolates, which formed a complex by 17 loci multilocus variable-number tandem-repeat analysis. [file spectrum.04115-25-s0001.pdf]

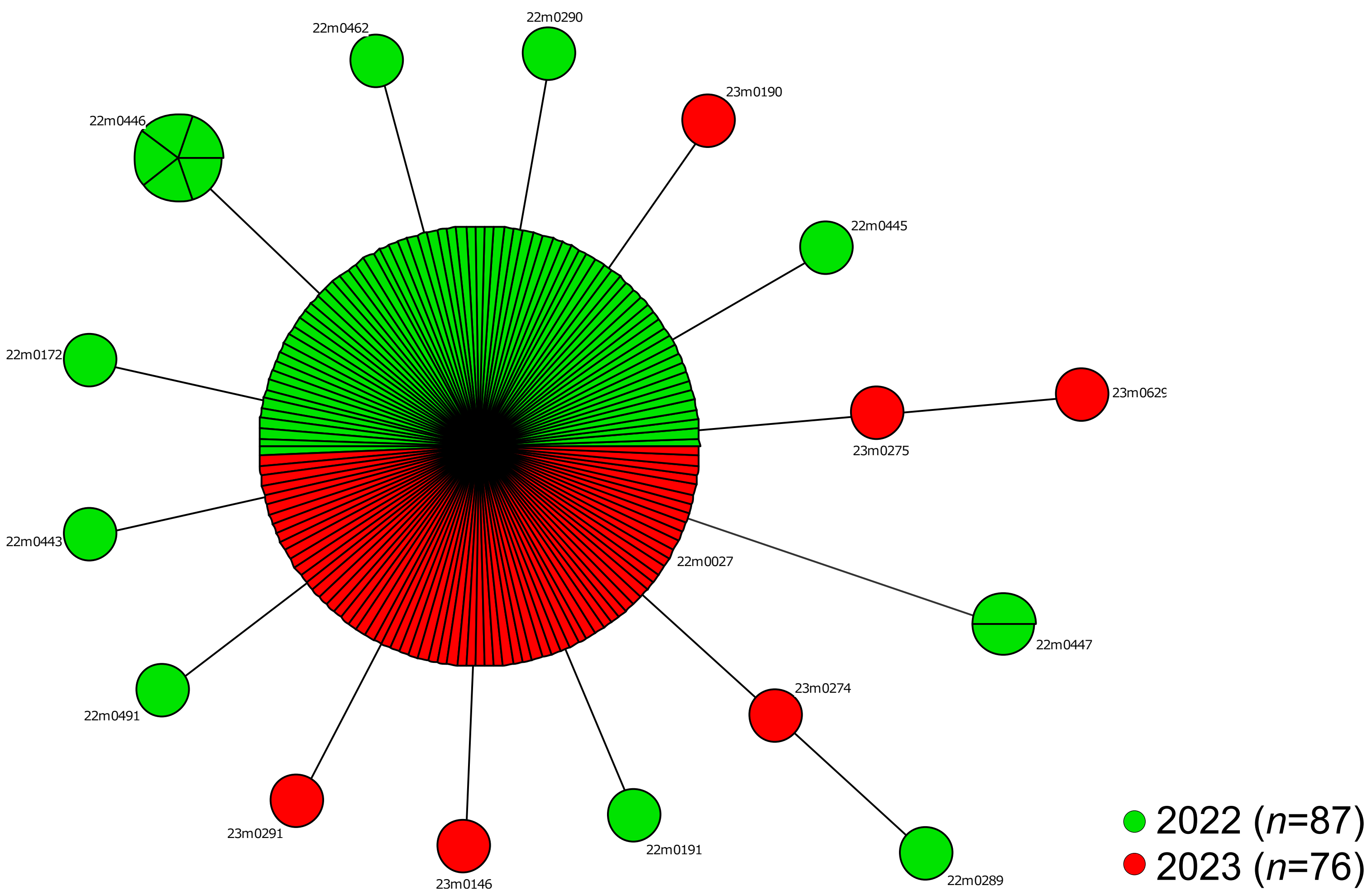

Fig. S1. Minimum spanning tree constructed from 163 Shiga toxin–producing *Escherichia coli* isolates, which formed a complex by 17 loci multilocus variable-number tandem-repeat analysis. Circles denote isolates; a line between circles indicates a single locus variant.
